# The environmental impact of diet in Latin American populations: a systematic review with meta-analysis

**DOI:** 10.1017/S1368980026102584

**Published:** 2026-05-04

**Authors:** Carolina Araya-Bastias, Josefa MF. Garzillo, Solange Parra-Soto, Jana Anderson, Duncan Lee, Jill P. Pell, Carlos A. Celis-Morales

**Affiliations:** 1 School of Health and Wellbeing, University of Glasgow, Glasgow, UK; 2 School of Cardiovascular and Metabolic Health, University of Glasgow, Glasgow, UK; 3 Center for Epidemiological Research in Nutrition and Health (NUPENS), University of São Paulo, Brazil; 4 Departamento de Nutrición y Salud Pública, Universidad del Bio-Bio – Sede Chillan, Chile; 5 School of Mathematics and Statistics, University of Glasgow, Glasgow, UK; 6 Human Performance Lab, Education, Physical Activity, and Health Research Unit, University Católica del Maule, Chile; 7 High-Altitude Medicine Research Centre (CEIMA), Universidad Arturo Prat, Chile

**Keywords:** Carbon footprint, Greenhouse gas emissions, Water footprint, Diet, Latin America

## Abstract

**Objective:**

To examine and synthesise data on the environmental impact of diet in Latin America.

**Design:**

A systematic review was conducted in April 2024, using Medline, Embase, Web of Science and Scopus databases, and updated in March 2025. We synthesised the evidence on the most reported environmental impact indicators. Meta-analysis was conducted to derive pooled estimates for individual countries of the mean dietary carbon and water footprints per person/per day; crude and energy-standardised (to 8368 kJ (2000 kcal)) values and stratified by dietary assessment method and sociodemographic variables.

**Setting:**

Latin America.

**Participants:**

Latin American populations.

**Results:**

Of the 2766 studies screened, 31 were included. Data on environmental impact of diet were reported for eight Latin American countries, with most coming from Brazil. Dietary water footprint ranged from 2078 in Chile to 3215 L/person/day/8368 kJ in Brazil. Dietary carbon footprint ranged from 2·1 to 7·3 kgCO_2_-equivalents/person/day/8368 kJ, in Peru and Argentina, respectively. The pooled standardised carbon footprint mean was 4·1 (95 % CI 2·6, 5·5, I^2^ = 100 %) kgCO_2_-equivalents/person/day/8368 kJ with no significant differences between dietary assessment method (*P* = 0·86). A higher carbon footprint was observed in individuals with higher education level and urban residence (*P* < 0·05).

**Conclusion:**

The available evidence suggested a wide variation in dietary environmental impact between Latin American countries, but a paucity of studies conducted in countries other than Brazil. Standardised approach to estimate the environmental impact of diet across the region and analytical perspectives in further research would support the development of country-relevant evidence-based public policies for sustainable diets in Latin America.

Food systems are central to the planetary health crisis. It accounts for approximately one-third of global greenhouse gas emissions (GHGE), drives biodiversity loss through land-use change and exerts substantial pressure on freshwater resources^(1–3)^. Current dietary patterns, particularly those characterised by high consumption of animal-source foods and ultra-processed products, are major contributors to these environmental impacts^(4,5)^. At the same time, diets are a leading determinant of non-communicable diseases^(6)^, placing food systems at the intersection of climate change, ecosystem degradation and population health^(7,8)^.

Environmental impacts of diets are typically quantified using life cycle assessment metrics, including GHGE expressed as carbon dioxide equivalents (CO₂eq), water footprint encompassing green, blue and grey water^(9,10)^. Additional indicators such as land use, eutrophication and acidification potentials capture nutrient runoff and ecosystem disruption^(2,11)^. Although these measures provide complementary insights into ecological pressures, most research has focused primarily on carbon footprints, often overlooking other environmental dimensions and regional specificities^(12,13)^.

Evidence from high-income countries shows that average diet-related emissions frequently exceed 3–6 kg CO₂eq per person per day, with animal-based dietary patterns consistently associated with substantially higher emissions, land requirements and water use compared with plant-based diets^(14–16)^. Modelling studies suggest that dietary shifts, alongside reductions in food waste and improvements in production efficiency, could reduce food-system emissions by up to 45 %^(17)^. However, these estimates are largely derived from Europe, North America and Australia, limiting their generalisability to regions with distinct food systems, agricultural structures and socioeconomic contexts.

Latin America is of particular relevance to the global sustainability agenda. The region is undergoing rapid nutrition transitions marked by increasing consumption of animal-source foods and ultra-processed products, while persistent undernutrition and food insecurity remain in vulnerable populations^(18,19)^. This coexistence of dietary excess and deprivation creates complex sustainability and equity challenges. Moreover, Latin America contains some of the world’s most biodiverse ecosystems, including the Amazon basin, and plays a critical role in global agricultural production and exports^(19–21)^. Accelerating deforestation, land degradation and climate vulnerability threaten both ecological integrity and long-term food security. Despite this strategic importance, evidence quantifying the environmental impacts of dietary patterns in Latin American populations remains fragmented and methodologically heterogeneous.

Robust, context-specific synthesis of this evidence is urgently needed to inform policies that align dietary guidelines, climate commitments and health equity objectives. Therefore, we conducted a systematic review and meta-analysis to synthesise available evidence on the environmental impacts of dietary patterns in Latin America. We aimed to quantify diet-related environmental footprints, examine methodological approaches used in the region and identify critical evidence gaps to support the development of sustainable, health-promoting food policies.

## Methods

We conducted a systematic review with meta-analysis of the published evidence on the environmental impact of dietary patterns in Latin American populations. The initial searches were conducted on April 3rd, 2024, and data extraction and analysis were completed over the following four months. An updated search and analysis occurred on March 4th, 2025. We followed the Preferred Reporting Items for Systematic Reviews and Meta-Analyses guidelines, and the full protocol for the systematic review was published in PROCEED (Reference 24-00274).

Searches were conducted using Medline, Embase, Web of Science and Scopus databases through Ovid. The search terms, detailed in online Supplemental Material 1, were selected after reviewing the titles and keywords used in known relevant papers. Reference lists of included articles were also scanned for additional relevant studies. Published studies were included in the systematic review if they measured at least one environmental impact indicator (EII) relevant to a country’s population diet, such as GHGE, carbon footprint, water footprint or ecological footprint. Eligible countries included Argentina, Belize, Bolivia, Brazil, Chile, Colombia, Costa Rica, Ecuador, El Salvador, Guatemala, Guyana, Honduras, Mexico, Nicaragua, Panama, Paraguay, Peru, Suriname, Uruguay and Venezuela. Eligible sources of food consumption data included national consumption surveys, household surveys or FAO Food Balance Sheets. We excluded studies that did not report results by individual country, as well as those focusing solely on the environmental impact of specific foods or meals rather than overall diet or dietary patterns. Studies based exclusively on modelling without any real-world data analysis were also excluded. No restrictions were placed on study design, date of publication or language.

Following the removal of duplicates, titles, then abstracts, were screened against the inclusion and exclusion criteria. Where abstracts lacked sufficient detail for assessment, full-text screening was undertaken. Two independent reviewers (CA-B and JMFG) screened and selected studies for eligibility, with any discrepancies resolved by a third reviewer (CC-M). This process was conducted using the online tool 2024 Covidence ©.

### Quality assessment

Most of the included studies used a cross-sectional design, so we assessed study quality using the Observational Study Quality Evaluation instrument^(22)^. The Observational Study Quality Evaluation tool has three versions tailored to cohort, case–control and cross-sectional studies. The cross-sectional version is an adaptation of the cohort version, containing 7 core items, 3 optional items and 4 non-scored questions that provide an overall evaluation of the study’s methodology. The specific criteria for each item are detailed in online Supplemental Material 2. In this review, we applied a 9-item Observational Study Quality Evaluation scale, with a maximum score of 9 stars. The overall score was then categorised into: low (1–3 stars), moderate (4–6 stars) and high (7–9 stars) quality. The tool was applied by two review authors (CA-B and CC-M) to each included study independently. Discrepancies in scoring were discussed to reach agreement.

### Data extraction

The two reviewers (CA-B and CC-M) extracted and tabulated key data from the included studies using Microsoft Excel: country and year of publication; sample size; characteristics of the study population; representativeness of the sampling frame; dietary assessment methods; EII; and methods for estimating footprint values, including Life Cycle Assessment (LCA) stages.

The LCA boundaries reported in the included studies were documented and categorised according to the following stages: food production to farm gate; to factory gate; to distribution point; to retail outlet; to kitchen; and to final disposal^(23)^.

All EII reported in the eligible studies were extracted as presented in the original publications. These included, but were not limited to, GHGE or carbon footprint, water footprint (green, blue and grey components, reported either separately or combined), land use, ecological footprint, fossil energy use, eutrophication potential and other indicators when available. For the purpose of quantitative synthesis, carbon and water footprints were identified a priori as the primary outcomes for meta-analysis due to their more frequent reporting and greater comparability across studies.

Where available, we extracted overall dietary EII as well as food group–specific contributions. We also extracted the environmental footprint estimates used for meat by type expressed per kg of product, given its established contribution to dietary environmental impacts^(24)^. Mean values and measures of dispersion (sd, se, interquartile range, or 95 % CI) were extracted in their original units. Mean dietary energy intake for the total sample was also recorded. When EII and energy intake were reported by sociodemographic subgroup (e.g., sex, age, education level, socioeconomic status or place of residence), these subgroup-specific data were extracted separately.

Extracted datasets were cross-checked between reviewers to ensure accuracy and completeness. Missing data required for meta-analysis (e.g., dispersion measures or sample sizes) were verified against the full text, supplementary materials or through contact with corresponding authors when necessary. We did not exclude values identified as extreme unless a clear reporting or calculation error was detected. When inconsistencies or implausible values were identified, they were checked against the original publication and clarified with study authors where possible.

### Data synthesis

Data from all the included studies were summarised in tables according to the most frequently reported EII: carbon footprint or GHGE, water footprint or freshwater consumption and ecological footprint.

Given the diversity of populations and methodological approaches across studies, we conducted meta-analyses of mean carbon and water footprints to generate pooled regional estimates and to examine potential differences according to dietary assessment method. Studies were eligible for inclusion in the meta-analyses if they met the following criteria:

- nationally representative sampling;
- dietary assessment based on food consumption surveys (e.g., 24-hour recalls or FFQ) or household food purchase surveys;
- environmental footprints reported per capita or per person per day; and
- availability of sample size, a measure of central tendency (mean or median) and a measure of dispersion (sd, se, interquartile range (IQR), or 95 % CI).

Given the expected heterogeneity in dietary patterns, data sources and LCA boundaries, a substantial degree of between-study variability was anticipated. When multiple publications were based on the same national survey and/or study population within a country, only one estimate was included in the meta-analysis. In such cases, the most recent study with the highest methodological quality score, as assessed using the Observational Study Quality Evaluation tool, was selected to avoid duplication and ensure methodological robustness. Pooled estimates of carbon footprint were presented as forest plots stratified by dietary assessment method (food consumption surveys *v*. food purchase surveys). Additional pooled estimates by sociodemographic characteristics were presented in tabular format. Water footprint estimates were summarised descriptively and, where appropriate, pooled estimates were presented in tables.

### Data analysis

EII were harmonised to common units to ensure comparability across studies. Carbon footprint values were expressed in kilograms of CO₂ equivalents (kg CO₂eq), water footprint in litres (L) and ecological footprint in square metres (m^2^), all standardised to a per person per day basis where appropriate. When studies reported results in alternative formats (e.g., per household, per year or per kilogram of food), values were converted to per person per day using the information provided in the original publication.

To account for differences in total energy intake between populations, EII were analysed both as crude values and after standardisation to an energy intake of 8368 kJ (2000 kcal) per day. This reference level corresponds to the mean adult energy recommendation and is commonly used as the daily reference intake for nutrition labelling.

For the meta-analysis, carbon and water footprint values were further converted to a common statistical scale using reported means and measures of dispersion (sd or se), allowing pooled means with 95 % CI.

Given that meat, particularly beef and other red meats, is a major contributor to diet-related environmental impacts, we additionally estimated population-level consumption of beef and red meat (including beef, pork, lamb and other ruminant meats) for studies included in the carbon footprint meta-analysis. Where specific consumption data were not directly reported, estimates were derived from the same study sources or calculated based on reported energy intake from beef and pork using data from the United States Department of Agriculture food composition database^(25,26)^.

Subgroup meta-analyses for carbon footprint (crude and energy-standardised) were conducted based on sociodemographic characteristics when reported by the study. Sociodemographic variables were categorised as follows: sex (male, female); socioeconomic status level (low, middle and high); education level (≤ 8, 9–12, > 12 schooling years); age groups (10–19, 20–30, 31–59, ≥ 60 years) and residential location (urban, rural).

Pooled estimates were calculated using the DerSimonian and Laird inverse-variance random-effects model, which accounts for between-study heterogeneity. Statistical heterogeneity was quantified using the I^2^ statistic. Analyses were performed using Stata version 18 (StataCorp), and statistical significance was defined as *P* < 0·05.

## Results

Our search identified 4268 records, of which 1502 were duplicates. After title and abstract screening, 2716 records were excluded. The most common reasons for exclusion at this stage were as follows: studies conducted outside Latin America; analyses focused on single food items or food products rather than overall dietary patterns; modelling studies without empirical dietary intake data; studies assessing environmental impacts unrelated to diet; and non-original research articles (e.g., reviews, editorials, or conference abstracts).

**Figure 1.**
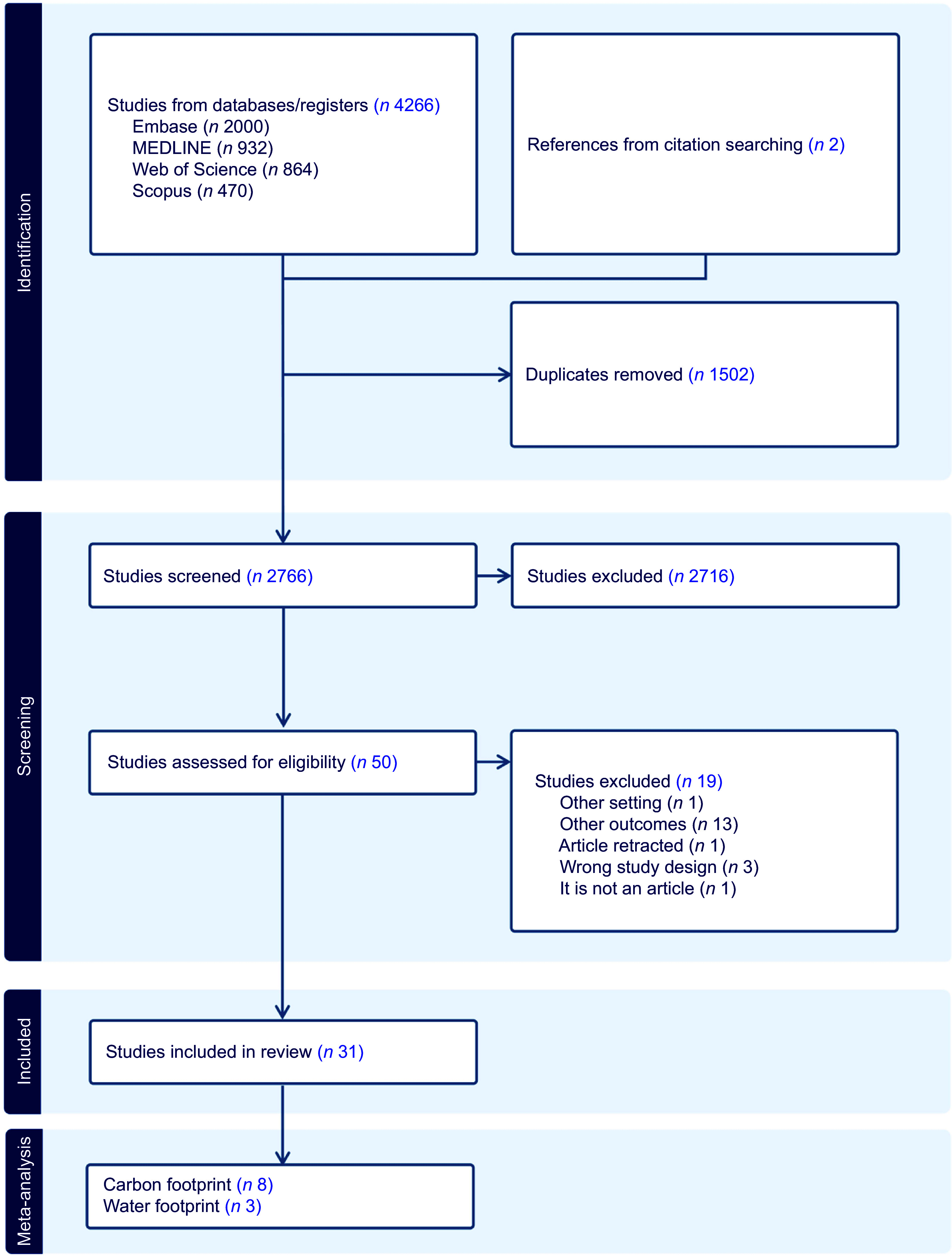
Preferred Reporting Items for Systematic Reviews and Meta-Analyses flow diagram of the identification, screening and selection process for included studies.

Of the 50 articles assessed in full text, 19 were excluded due mostly to lack of relevant EII, insufficient methodological detail, non-representative populations, or absence of extractable data or wrong study design^(27–45)^. Consequently, 31 studies were included in the systematic review, of which 8 and 3 met criteria for meta-analysis of carbon and water footprint, respectively^(46–76)^ (Figure 1).

The studies included in our review reported the environmental impact of diets across eight Latin American countries, with the majority conducted in Brazil (*n* 13), followed by Mexico (*n* 6), Argentina (*n* 4), Chile (*n* 3) and Peru (*n* 2). Only one study each was conducted in Colombia, Costa Rica and Guatemala, and none in the remaining 12 Latin American countries. Most studies were published recently, between 2017 and 2024.

The most frequently reported EII were GHGE or carbon footprint. In most studies, these terms were used interchangeably and expressed as kg CO₂eq, reflecting total climate change impact across the food life cycle. For consistency, we refer to both measures collectively as carbon footprint in the quantitative synthesis. Water footprint was the second most commonly reported indicator. In contrast, other EII, such as land occupation, fossil energy use, eutrophication potential and biodiversity-related metrics, were rarely assessed.

The majority of studies utilised dietary data from national surveys that measured individual food consumption using 24-hour dietary recall (24HR) (*n* 10), FFQ (*n* 10) or household food purchase records (*n* 11). Additionally, half of the articles analysed data by sociodemographic subgroups, such as sex, age group, education level or socioeconomic status (Table 1). In the quality assessment, 28 of the 31 studies were classified as moderate or high quality, while three studies were classified as low quality (online Supplemental Material 1 and Table 1).

**Table 1.** Characterisation and quality assessment level of included studies (*n* 31)

Authors, year, reference	Country	Study design	Analytical sample characteristics	National representativeness (Yes/No)	Dietary assessment method	Source of information	Environmental indicators (unit)	Sociodemographic variables	Quality assessment level
Arrieta E.M *et al.*, 2018,^(50)^	Argentina	Population survey	N/R	Yes	Household survey, food purchase	The National Survey of Household Income and Expenditure 2012/2013	GHGE (kg CO_2_eq/person/day)	N/A	Moderate
Arrieta E.M *et al.*, 2021,^(49)^	Argentina	Population survey	21 547 households	Yes	Household survey, food purchase	The National Survey of Household Income and Expenditure 2017/2018	GHGE (kgCO_2_eq/day/2000 kcal), Total land occupation (m^2^/day/2000 kcal), Cropland demand (m^2^/day/2000 kcal), Fossil energy use (Mj/day/2000 kcal), Freshwater consumption (L/day/2000 kcal), and Eutrophying emissions (gPO4eq/day/2000 kcal)	Socioeconomic level	High
Arrieta E.M *et al.*, 2022,^(48)^	Argentina	Population survey	N/R	Yes	Household survey, food purchase	The National Survey of Household Income and Expenditure 2017/2018	GHGE (Mt CO_2_eq/year), Total land occupation (Mha/year), Cropland demand (Mha/year), Fossil energy use (Pj/year), Freshwater consumption (Km3/year), and Eutrophying emissions (MtPO4e/year)	N/A	Moderate
Telis M.S *et al.*, 2023,^(72)^	Argentina	Cross-sectional	170 Argentinian Nutrition professionals, Female 97·6 %, 22–64 years old	No	A self-administered online questionnaire, FFQ	Primary	Water footprint (L/person/day)	N/A	Moderate
Travassos G.F *et al.*, 2020,^(73)^	Brazil	Population survey	24 152 participants, Female 55 %, 18 to ≥60 years old	Yes	2 Non-consecutive 24HR dietary recalls	The National Dietary Survey, part of The 2008/2009 Brazilian Household Budget Survey (POF in Portuguese)	Carbon footprint (gCO_2_eq/capita/day), Water footprint (L/capita/day), and Ecological footprint (m^2^/capita/day)	Gender, race, age, education level, household status, region, urban or rural area	High
Vale *et al.*, 2021,^(74)^	Brazil	Ecological study	102 072 adolescents, Female 51·7 %, 13 to 15 years old	No	FFQ	The 2015 National Adolescent Health Survey (PeNSE in Portuguese)	Water footprint (L/kg Food group)	Brazilian federation unit and geographic macro-region.	Moderate
Garzillo J.M.F *et al.*, 2021,^(59)^	Brazil	Population survey	34 003 participants, Female 48 %, 10 to ≥60 years old	Yes	2 Non-consecutive 24HR dietary recalls	The National Dietary Survey, part of the 2008/2009 Brazilian Household Budget Survey (POF in Portuguese)	Carbon footprint (gCO_2_eq/person/day) and (gCO_2_eq/2000 kcal)	Gender, age, education level, family income per capita, macro-region, and federative unit	High
Aguiar D.R *et al.*, 2021,^(46)^	Brazil	Population survey	N/R	Yes	Household survey, food purchase	The 2008/2009 Brazilian Household Budget Survey (POF in Portuguese)	GHGE (kgCO_2_eq/person/year)	Region, capitals (states), income level, and place of residency.	Moderate
da Silva *et al.*, 2021,^(56)^	Brazil	Time-series study	N/R	Yes	Household survey, food purchase	5 Brazilian Household Budget Surveys (1987–1988, 1995–1996, 2002–2003, 2008–2009, 2017–2018)	GHGE (CO_2_eq/1000 kcal/year), Water footprint (L/1000 kcal/year), Ecological footprint (m^2^/1000 kcal/year)	N/A	High
Bezerra I.N. *et al.*, 2022,^(52)^	Brazil	Population survey	22 780 participants, 20 to 60 years old	No	2 Non-consecutive 24HR dietary recalls	The National Dietary Survey, part of the 2017/2018 Brazilian Household Budget Survey (POF in Portuguese)	Carbon footprint (gCO_2_eq/person/day)	Age, region, household income per capita	High
Hatjiathanassiadou *et al.*, 2022,^(64)^	Brazil	Cross-sectional	411 participants, Female 57·9 %, 20 to ≥60 years old	No	FFQ and one 24HR dietary recall	Proyect: ‘Food Insecurity, Health and Nutrition Conditions in an Adult and Elderly Population of a Capital in the Northeast Region of Brazil: BRAZUCA Natal/RN Study’	Carbon Footprint (gCO_2_eq/person/day), Water Footprint (L/person/day), Ecological Footprint (m^2^/person/day) and all adjusted to 1000 kcal	Gender, age, ethnicity, educational level, family income	High
Garzillo J.M.F *et al.* (a), 2022,^(60)^	Brazil	Population survey	32 853 participants, Female 52 %, 10 to ≥60 years old	Yes	2 Non-consecutive 24HR dietary recalls	The National Dietary Survey, part of the 2008/2009 Brazilian Household Budget Survey (POF in Portuguese)	Carbon footprint (gCO_2_eq/1000 kcal); Water footprint (L/1000 kcal)	N/A	High
Garzillo J.M.F *et al.* (b), 2022,^(61)^	Brazil	Population survey	32 886, Female 48 %, 10 to ≥60 years old	Yes	2 Non-consecutive 24HR dietary recalls	The National Dietary Survey, part of the 2008/2009 Brazilian Household Budget Survey (POF in Portuguese)	Carbon footprint (gCO_2_eq/person/day, Water footprint (L/person/day)	N/A	High
Silva V. *et al.*, 2023,^(71)^	Brazil	Population survey	843 participants from Brasilia, Female 53 %, ≥ 24 to ≤65 years old	No	2 Non-consecutive 24HR dietary recalls	The National Dietary Survey, part of the 2017/2018 Brazilian Household Budget Survey (POF in Portuguese)	GHGE (tCO_2_eq/day), (kgCO_2_eq/kg Food), and (kgCO_2_eq/person/day)	Gender, age, household income, per capita income, rural or urban area	Moderate
da Cruz G.L, 2024,^(55)^	Brazil	Population survey	57 920 households	Yes	Household survey, food purchase	The National Dietary Survey, part of the 2017/2018 Brazilian Household Budget Survey (POF in Portuguese)	Carbon footprint (gCO_2_eq/person/day), Water footprint (L/person/day)	N/A	Moderate
Barbosa A.M. *et al.*, 2024,^(51)^	Brazil	Population survey	N/R	Yes	N/R	The National Dietary Survey, part of the 2017/2018 Brazilian Household Budget Survey (POF in Portuguese)	Water footprint (N/R unit)	N/A	Low
Verly E. *et al.*, 2024,^(76)^	Brazil	Population survey	33 859 participants ≥25 years old	Yes	2 Non-consecutive 24HR dietary recalls	The National Dietary Survey, part of the 2017/2018 Brazilian Household Budget Survey (POF in Portuguese)	Global warming potential (kgCO_2_eq); Land occupation (ha-year arable); Land transference (ha-year arable); Fossil energy use (MJ); Bluewater use (L); Freshwater acidification (kg SO2eq); Terrestrial acidification (kg SO2eq); Freshwater eutrophication (kg PO4– Peq); Marine eutrophication (kg Neq); Freshwater ecotoxicity (CTUe); Ozone layer depletion (kg CFC11eq)	N/A	Moderate
Franco C.C. *et al.*, 2022,^(58)^	Chile	Population survey	3 365 700 households	Yes	Household survey, food purchase	VIII Household Budget Survey 2017	Carbon footprint (kgCO_2_/person/day), Water footprint (L/person/day)	Socioeconomic level	Moderate
Gormaz T. *et al.*, 2022,^(62)^	Chile	Population survey	4920 participants, ≥2 years old	Yes	FFQ	The 2010 National Food Consumption Survey of Chile (ENCA in Spanish)	Carbon footprint (kgCO_2_/person/day), Water footprint (L/day/person)	Sex, socioeconomic level, age group, and macrozone location	High
Gutiérrez S. *et al.*, 2023,^(63)^	Chile	Population survey	4676, Female 62 %, ≥2 years old	Yes	FFQ	The 2010 National Food Consumption Survey of Chile (ENCA in Spanish)	GHGE (kgCO_2_eq/person/day)	Gender, age, socioeconomic level, rural or urban area, macrozones location	Moderate
Parra-Orobio B.A. *et al.*, 2023,^(70)^	Colombia	Cross-sectional	101 households from Fátima neighbourhood	No	Household survey, food purchase	Primary	Water (green) footprint (m3/month)	Socioeconomic level	Low
Aljahdali A.A. *et al.*, 2023,^(47)^	Costa Rica	Case-control study	3634 (1817 nonfatal MI/ 1817 controls), Female 25 %, 20–70 years old	No	FFQ	Data from the Costa Rica Heart Study	GHGE (kgCO_2_eq/year)	Education level, income, marital status	Moderate
Escobar A.G. *et al.*, 2024,^(57)^	Guatemala	Cross-sectional	2082 women adolescents, 10 to 19 years old	No	One 24HR dietary recall	Data from the follow-up study of the impact of the biofortified bean variety SMN39 (Phaseolus vulgaris L) associated with agricultural and nutritional education (Estudio de control del impacto de frijol biofortificado variedad SMN39 (Phaseolus vulgaris L) asociado a la educación agrícola y nutricional in Spanish)	GHGE (kgCO_2_eq/person/day)	N/A	Low
Lares-Michel M. *et al.* (a), 2021,^(65)^	Mexico	Cross-sectional	237 adults from Jalisco, 18 to 74 years old	No	One 24HR dietary recall	Primary and from the 2018 Mexican National Health and Nutrition Survey (ENSANUT in Spanish)	Water footprint (L/g ingredients of preparation) and (L/person/day)	N/A	Moderate
Lares-Michel M. *et al.* (b), 2021,^(67)^	Mexico	Cross-sectional	395 participants, Female 64 %, 18 to 74 years old	No	FFQ	Primary	Water footprint (L/person/day)	N/A	High
López-Olmedo N. *et al.*, 2022,^(69)^	Mexico	Population survey	20 298 participants, ≥12 years old	Yes	FFQ	The 2018 Mexican National Health and Nutrition Survey (ENSANUT in Spanish)	GHGE (kgCO_2_eq/capita/day)	Gender, age, educational level, socioeconomic level, region, speaking an indigenous language	High
Curi-Quinto K *et al.*, 2022,^(54)^	Mexico	Population survey	2438 participants, 18 to 59 years old	Yes	FFQ	The 2012 Mexican National Health and Nutrition Survey (ENSANUT in Spanish)	Carbon footprint (kg CO2e/capita/day), Blue water footprint (L/capita/day), Land use (m^2^/capita/day), Biodiversity loss (potential number of species lost × 10^10^/capita/day)	Gender, age, socioeconomic level, educational level, ethnicity, rural or urban area, and region	High
Lares-Michel M. *et al.*, 2022,^(66)^	Mexico	Cross-sectional	400 participants from Guadalajara, Female 55 %, 18 to 74 years old	No	FFQ	Primary	Water footprint (L/day)	N/A	High
Canto‑Osorio F. *et al.*, 2024,^(53)^	Mexico	Time-series study	11 051 in 1989 to 88 398 in 2020	Yes	Household survey, food purchase	Data from 16 Mexico’s National Income and Expenditure Survey (1989; 1992; 1994; 1996; 1998; 2000; 2002; 2004; 2006; 2008; 2010; 2012; 2014; 2016; 2018; 2020)	GHGE per adult equivalent per day (kgCO_2_eq/ad-eq/day)	Education level and urbanicity.	High
Vazquez-Rowe I. *et al.*, 2017,^(75)^	Peru	Population survey	36 234 participants	Yes	Household survey, food purchase	The 2012 National survey performed by the Peruvian Statistics Unit (ENAPREF in Spanish)	GHGE (tCO_2_eq/day), (kgCO_2_eq/kg Food), and (kgCO_2_eq/ capita/year)	Geographic and socioeconomic scenarios	Moderate
Larrea-Gallegos G. *et al.*, 2020,^(68)^	Peru	Population survey	36 234 participants	Yes	Household survey, food purchase	The 2012 National survey performed by the Peruvian Statistics Unit (ENAPREF in Spanish)	GHGE (kgCO_2_eq/capita/year)	24 cities	Moderate

N/A: not applicable; N/R: data not reported/available; GHGE: Greenhouse Gas Emissions; CO_2_eq: Carbon Dioxide equivalents; PO4eq: Phosphate equivalents; CTUe: Comparative toxic units; SO2eq: Sulphur dioxide equivalents; Neq: Nitrogen equivalent; CFC11eq: Chlorofluorocarbon-11 equivalent.

### Carbon footprint or greenhouse gas emissions

The carbon footprint or GHGE values were reported for 24 of the 31 studies (online supplementary material, Supplemental Table S3.1). According to the most recent data, the energy-standardised mean for carbon footprint of diets ranged from 2·1 to 7·3 kg CO_2_eq/person/day/8368 kJ in Peru and Argentina, respectively. Of the different foods investigated, beef made the largest contribution to the total dietary carbon footprint, ranging from 15 % to 87 %, while pulses made the lowest contribution at approximately 1 %. The mean carbon footprint estimated per kilogram of beef ranged from 28·6 kg CO₂eq in Peru to 56·1 kg CO₂eq in Mexico. The most commonly reported LCA investigated were from food production to distribution point (*n* 5) and from food production to the kitchen (*n* 6), while two studies from Brazil and Guatemala did not specify the LCA stage considered in estimating EII. Additionally, 13 studies reported carbon footprint estimates stratified by sociodemographic variables. In general, diet-related carbon footprint was higher among men, middle-aged adults and individuals with higher educational level and socioeconomic status.

### Water footprint or freshwater consumption

Nineteen studies reported water footprint or freshwater consumption, with four studies exclusively reporting blue water footprint and one study reporting green water footprint. The most recent and nationally representative water footprint means for country-specific diets reported were 2078 L/person/day in Chile and 3215 L/person/day in Brazil. Beef made the largest contribution to water use (32–61 %), while the lowest contributors were vegetables, seafood and pulses, with contributions ranging from 0 % to 4 %. Most studies included water used in kitchen processes (LCA stage: 5, from production to consumption/preparation) as part of their EII estimation. The water footprint for beef ranged from 12 105 to 22 387 L/kg. Six studies reported this EII by sociodemographic factors and reported a higher water footprint for men (online supplementary material, Supplemental Table S3.2).

### Ecological footprint

Ecological footprint values were reported by three studies that used Brazilian data (online supplementary material, Supplemental Table S3.3). The full diet energy-standardised means reported for ecological footprint were 22·8, 28·6, and 85·2 m^2^/ person/day/8368 kJ. Beef made the highest contribution to ecological footprint, while vegetables made the lowest. Sociodemographic subgroup analyses were reported by one study which found a higher ecological footprint for men, for individuals with low income and educational level, and living in urban areas.

### Meta-analysis

**Figure 2.**
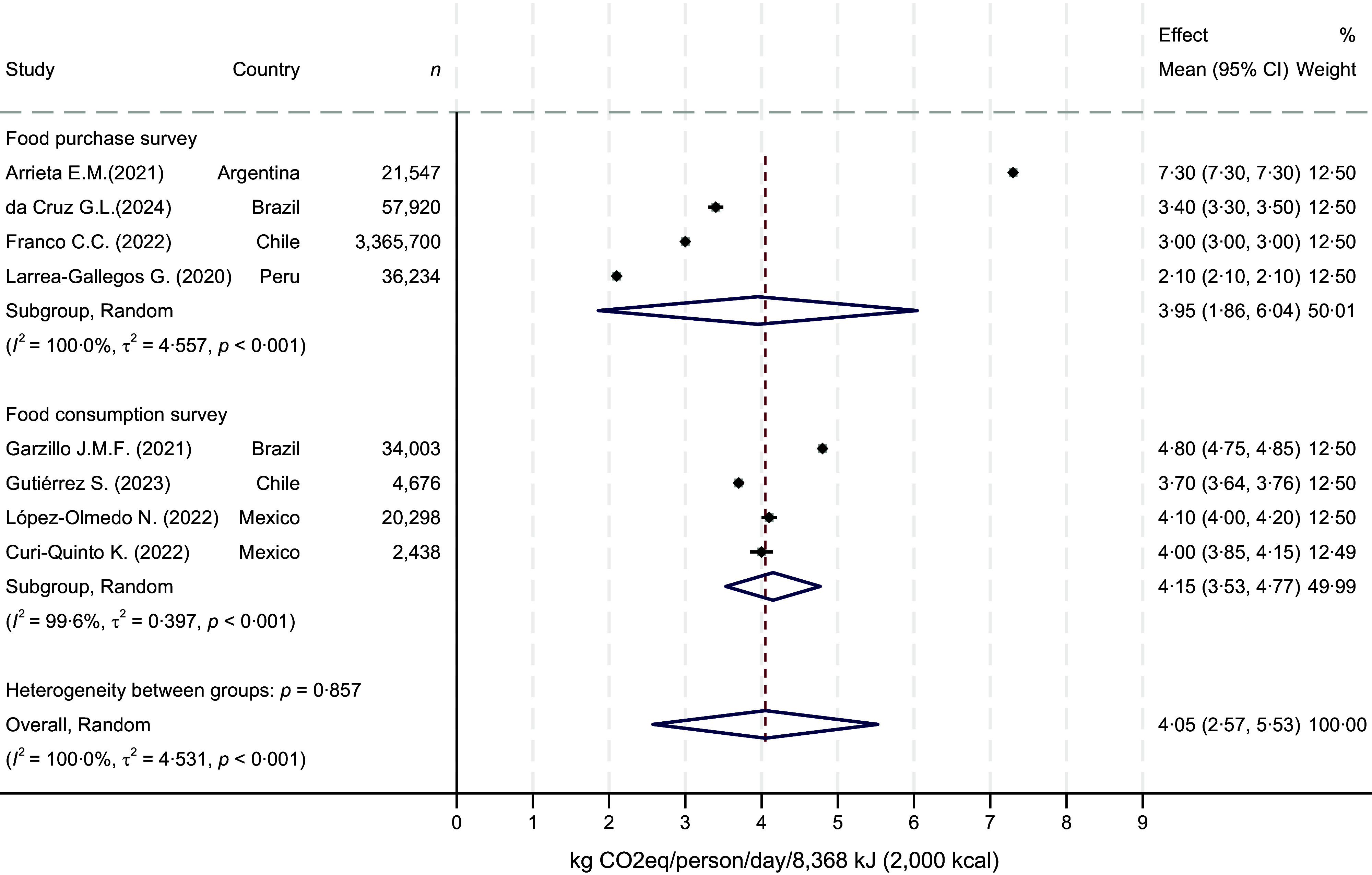
Forest plot of the meta-analysis showing the energy-standardised carbon footprint by dietary assessment method in Latin American countries. *Notes:* CO2 eq: Carbon dioxide equivalents. Food Purchase Survey (*n* 4) and Food Consumption Survey (*n* 4). Weight and between-subgroup heterogeneity test are from random-effects model.

Eight studies were included in meta-analyses of the energy-standardised mean carbon footprint stratified by dietary assessment method: four used food purchase data and four used food consumption surveys (Figure 2). The pooled energy-standardised carbon footprint mean was 4·1 (95 % CI 2·6, 5·5, I^2^ = 100 %) kgCO_2_eq/person/day/8368 kJ, which was similar to the pooled crude carbon footprint available in the online supplementary material, Supplemental Figure S3.1. The pooled carbon footprint showed greater variability within countries when derived from food purchase data compared to consumption surveys. However, the difference between dietary assessment methods was not statistically significant (*P* = 0·86).

Argentina and Brazil had the highest carbon footprint related to red meat consumption, and Chile and Peru had the lowest (online supplementary material, Supplemental Table S3.4). The meta-analysis of crude and energy-standardised carbon footprint means by sociodemographic subgroups revealed some differences. Crude carbon footprint values were significantly higher among men, individuals with the highest socioeconomic status, those with higher education levels, residents of urban areas and individuals aged 20–30 years. However, after energy standardisation, only differences by education level and place of residence remained significant (Tables 2 and 3). Brazil had a higher water footprint than Chile (Table 4). This difference became statistically significant after standardisation for energy intake (*P* < 0·01).

**Table 2.** Meta-analysis of crude mean carbon footprint by sociodemographic variables

	Sex												
	Male			Female									
Studies (country, lead author (year))	Mean	95 % CI	Weight (%)	Mean	95 % CI	Weight (%)							
Brazil, Garzillo J.M.F. (2021)	5·1	5·0, 5·1	25·0	3·9	3·9, 3·9	25·0							
Mexico, López-Olmedo N. (2022)	4·4	4·4, 4·4	25·0	3·4	3·4, 3·4	25·0							
Subgroup, random	4·8	4·1, 5·4	50·0	3·7	3·2, 4·2	50·0							
Overall, random	4·2	3·6, 4·9											
Overall, I^2^	100 %												
Between subgroups	6·8^*^												

	Socioeconomic status												
	Low		Weight (%)	Middle		Weight (%)	High		Weight (%)				
Argentina, Arrieta E.M. (2021)	6·0	6·0, 6·0	6·7	9·1	9·1, 9·1	6·7	11·6	11·6, 11·6	6·7				
Brazil, Garzillo J.M.F. (2021)	4·1	4·1, 4·1	6·7	4·5	4·5, 4·5	6·7	4·9	4·9, 4·9	6·7				
Chile, Franco C.C. (2022)	2·6	2·6, 2·6	6·7	3·5	3·5, 3·5	6·7	4·5	4·5, 4·5	6·7				
Mexico, López-Olmedo N. (2022)	3·3	3·3, 3·3	6·7	3·8	3·8, 3·8	6·7	4·5	4·5, 4·5	6·7				
Peru, Vazquez-Rowe I. (2017)	1·5	1·5, 1·5	6·7	2·5	2·4, 2·5	6·7	3·6	3·6, 3·6	6·7				
Subgroup, random	3·5	2·9, 4·1	33·3	4·7	4·3, 5·1	33·3	5·8	5·3, 6·3	33·3				
Overall, random	4·7	4·3, 5·1											
Overall, I^2^	100 %												
Between subgroups	36·6^*^												

	Education level (schooling years)												
	≤ 8 years		Weight (%)	9–12 years		Weight (%)	> 12 years		Weight (%)				
Brazil, Garzillo J.M.F. (2021)	4·3	4·3, 4·3	16·7	4·8	4·8, 4·8	16·7	4·9	4·9, 4·9	16·7				
Mexico, López-Olmedo N. (2022)	3·5	3·5, 3·5	16·7	4·3	4·3, 4·3	16·7	4·9	4·9, 4·9	16·7				
Subgroup, random	3·9	3·1, 4·7	33·3	4·5	4·1, 5·0	33·3	4·9	4·9, 5·0	33·3				
Overall, random	4·4	4·0, 4·9											
Overall, I^2^	100 %												
Between subgroups	8·8^*^												

	Age group (years)												
	10–19		Weight (%)	20–30		Weight (%)	31–59		Weight (%)	≥ 60			Weight (%)
Brazil, Garzillo J.M.F. (2021)	4·4	4·4, 4·4	12·5	4·8	4·8, 4·8	12·5	4·6	4·6, 4·6	12·5	3·9		3·9, 3·9	12·5
Mexico, López-Olmedo N. (2022)	3·6	3·6, 3·6	12·5	4·5	4·5, 4·5	12·5	4·0	4·0, 4·0	12·5	3·4		3·4, 3·4	12·5
Subgroup, random	4·0	3·2, 4·7	25·0	4·6	4·3, 4·9	25·0	4·3	3·7, 4·8	25·0	3·6		3·1, 4·2	25·0
Overall, random	4·1	3·8, 4·5											
Overall, I^2^	100 %												
Between subgroups	10·0^*^												

	Location												
	Urban		Weight (%)	Rural		Weight (%)							
Brazil, Travassos G.F. (2020)	6·7	6·6, 6·8	16·7	7·0	7·0, 7·2	16·6							
Mexico, López-Olmedo N. (2022)	4·1	4·1, 4·1	16·7	3·3	3·3, 3·3	16·7							
Mexico, Curi-Quinto K. (2022)	4·1	4·1, 4·2	16·7	2·9	2·9, 2·9	16·7							
Subgroup, random	4·9	4·8, 5·1	50·1	4·4	4·0, 4·7	49·9							
Overall, random	4·7	4·2, 5·1											
Overall, I^2^	100 %												
Between subgroups	8·9^*^												

Values represent the carbon footprint expressed as kg CO2 (carbon dioxide) equivalents/person or capita/day (mean (95 % CI)).

* *P* < 0·05.

**Table 3.** Meta-analysis of energy-standardised mean carbon footprint by sociodemographic variables

	Sex												
	Male			Female									
Studies (country, lead author (year))	Mean	95 % CI	Weight (%)	Mean	95 % CI		Weight (%)						
Brazil, Garzillo J.M.F. (2021)	4·9	4·9, 4·9	25·0	4·6	4·6, 4·6		25·0						
Mexico, López-Olmedo N. (2022)	4·0	4·0, 4·0	25·0	4·3	4·3, 4·3		25·0						
Subgroup, random	4·4	3·5, 5·3	50·0	4·5	4·1, 4·8		50·0						
Overall, random	4·5	4·1, 4·8											
Overall, I^2^	100 %												
Between subgroups	0·0												

	Socioeconomic status												
	Low		Weight (%)	Middle			Weight (%)	High		Weight (%)			
Argentina, Arrieta E.M. (2021)	7·1	7·1, 7·1	6·7	7·4	7·4, 7·4		6·7	7·3	7·3, 7·3	6·7			
Brazil, Garzillo J.M.F. (2021)	4·4	4·4, 4·4	6·7	4·8	4·8, 4·8		6·7	5·1	5·1, 5·1	6·7			
Chile, Franco C.C. (2022)	2·8	2·8, 2·8	6·7	3·0	3·0, 3·0		6·7	3·2	3·2, 3·2	6·7			
Mexico, López-Olmedo N. (2022)	3·5	3·5, 3·5	6·7	4·1	4·1, 4·1		6·7	4·7	4·7, 4·7	6·7			
Peru, Vazquez-Rowe I. (2017)	2·0	2·0, 2·0	6·7	2·4	2·4, 2·4		6·7	2·9	2·9, 2·9	6·7			
Subgroup, random	4·0	3·3, 4·6	33·3	4·3	3·5, 5·2		33·3	4·7	3·8, 5·5	33·3			
Overall, random	4·3	4·0, 4·6											
Overall, I^2^	100 %												
Between subgroups	1·7												

	Education level (schooling years)												
	≤ 8 years		Weight (%)	9–12 years			Weight (%)	> 12 years		Weight (%)			
Brazil, Garzillo J.M.F. (2021)	4·7	4·7, 4·7	16·7	4·9	4·9, 4·9		16·7	5·2	5·1, 5·2	16·7			
Mexico, López-Olmedo N. (2022)	3·9	3·9, 3·9	16·7	4·4	4·4, 4·4		16·7	5·0	5·0, 5·0	16·7			
Subgroup, random	4·3	3·5, 5·0	33·3	4·6	4·1, 5·1		33·3	5·1	4·9, 5·2	33·3			
Overall, random	4·6	4·2, 5·1											
Overall, I^2^	100 %												
Between subgroups	6·2^*^												

	Age group (years)												
	10–19		Weight (%)	20–30			Weight (%)	31–59		Weight (%)	≥ 60		Weight (%)
Brazil, Garzillo J.M.F. (2021)	4·4	4·4, 4·4	12·5	4·8	4·8, 4·8		12·5	4·9	4·9, 4·9	12·5	4·8	4·8, 4·8	12·5
Mexico, López-Olmedo N. (2022)	3·9	3·9, 3·9	12·5	4·2	4·2, 4·2		12·5	4·2	4·2, 4·2	12·5	4·3	4·3, 4·3	12·5
Subgroup, random	4·1	3·6, 4·6	25·0	4·5	3·9, 5·1		25·0	4·6	3·8, 5·3	25·0	4·5	4·0, 5·0	25·0
Overall, random	4·4	4·2, 4·7											
Overall, I^2^	100 %												
Between subgroups	1·9												

	Location												
	Urban		Weight (%)	Rural			Weight (%)						
Brazil, Travassos G.F. (2020)	8·6	8·6, 8·7	16·7	8·4	8·2, 8·5		16·4						
Mexico, López-Olmedo N. (2022)	4·3	4·3, 4·3	16·7	3·4	3·4, 3·4		16·7						
Mexico, Curi-Quinto K. (2022)	4·3	4·3, 4·3	16·7	3·2	3·2, 3·2		16·7						
Subgroup, random	5·7	5·5, 5·9	50·1	4·8	4·6, 5·1		49·9						
Overall, random	5·4	4·9, 5·9											
Overall, I^2^	100 %												
Between subgroups	26·6^*^												

Values represent the carbon footprint expressed as kg CO2 (carbon dioxide) equivalents/person or capita/day standardised by 8368 kJ (2000 kcal) (mean (95 % CI)).

* *P* < 0·05.

**Table 4.** Meta-analysis of crude/energy-standardised mean water footprint

	Crude			Energy-standardised^*^		
Studies (lead author (year), dietary method)	Mean	95 % CI	Weight (%)	Mean	95 % CI	Weight (%)
Brazil						
Garzillo J.M.F. (a) (2022), Food consumption survey	4385·6	4336·2, 4435·0	33·3	4614	4562, 4666·0	33·3
da Cruz G.L. (2024), Food purchase survey	1963·8	1910·1, 2017·5	33·3	3215·4	3127·4, 3303·4	33·3
Subgroup, random	3174·7	801·4, 5548·1	66·7	3915·2	2544·6, 5285·8	66·7
Chile						
Franco C.C. (2022), Food purchase survey	2390·3	2389·7, 2391·0	33·4	2078·3	2077·8, 2079·0	33·4
Overall, random	2913·2	1715·8, 4110·6		3302·5	1495·3, 5109·7	
Between subgroups	0·4			6·9		
*P*-value	0·517			0·01		

Values represent the water footprint expressed as litres/person or capita/day (mean (95 % CI)).

* Values standardised by 8368 kJ (2000 kcal).

## Discussion

This systematic review highlights the limited and uneven evidence on the environmental impacts of diets in Latin America. Data were available for only eight of twenty countries in the region, with a strong concentration of studies conducted in Brazil. Most investigations focused on carbon and water footprints, while other environmental dimensions such as land use, eutrophication potential, fossil energy demand and biodiversity loss were rarely assessed. Across available data, Argentina and Brazil tended to report the highest dietary environmental footprints, whereas Peru consistently showed lower estimates. Beef was the dominant contributor to both carbon and water footprints, while pulses, vegetables and seafood generally contributed least. Higher environmental impacts were observed among individuals with higher educational level and urban residence. Studies based on food purchase surveys showed greater heterogeneity than those using individual consumption data.

Differences in environmental footprints across countries appear to be strongly driven by variations in dietary patterns, particularly the consumption of animal-source foods. Argentina, which reported the highest carbon footprint, has one of the highest levels of beef consumption in the region (120·7 g/person/day), substantially exceeding intake levels reported in Chile and Peru^(49,63,68)^. The environmental burden associated with this consumption is further amplified by country-specific production systems. For example, the carbon footprint of beef production in Argentina has been estimated at 41 kgCO₂eq/kg^(49)^, exceeding the global mean of 29 kgCO₂eq/kg reported in a systematic review by Clune *et al.* (2017)^(77)^. That review also documented a wide global range for beef production emissions, from approximately 9 to over 60 kgCO₂eq/kg, depending on production system, feed composition, land-use change, and methodological boundaries. Within this global context, the Argentine estimate lies above the reported mean and toward the higher end of pasture-based production systems, where enteric methane emissions and land occupation contribute substantially to total GHGE.

Similarly, Brazil reported the highest water footprint among the two countries with available data, a finding that can be largely attributed to high beef intake combined with grazing-based production systems, which are associated with particularly elevated water footprints^(60,78)^. Together, these results reinforce the central role of livestock production in shaping multiple environmental dimensions of dietary impact in Latin America, both at the consumption and production levels.

Dairy products also contributed meaningfully to overall dietary footprints, although their relative importance varied between countries^(50,53,55,58,61–63,69,75)^. In Chile, dairy accounted for a substantial proportion of both carbon and water footprints, whereas in Peru its contribution was considerably smaller^(58,62,63,75)^. Differences in consumption levels likely explain part of this variation. In some countries, intake appears to exceed levels recommended in reference dietary frameworks. National policies may further influence consumption patterns; for example, in Chile milk-based products included in supplementary feeding programmes may increase population-level intake^(79)^. Although dairy has a lower per-kilogram impact than beef, high levels of consumption can substantially increase national dietary footprints^(5)^.

The pooled energy-standardised dietary carbon footprint for the region (4·1 kgCO₂eq/person/day) substantially exceeds the levels associated with the EAT-Lancet reference diet and the Uruguayan dietary guidelines^(80,81)^. This difference can largely be explained by the higher consumption of meat and lower intake of plant-based foods across many Latin American countries, where animal-source food consumption – including meat, fish and seafood, and eggs – is considerably higher in several countries. For instance, mean animal foods intake from these sources reaches 204 g/person/day in Brazil and 233 g/person/day in Argentina, while Chile and Peru report lower, but still elevated, intakes of 102 and 107 g/person/day, respectively^(48,63,68,73)^. These levels still exceed the EAT-Lancet recommendation and the Uruguayan dietary guidelines of 84 and 100 g/person/day, respectively^(80,81)^. These findings align with global evidence indicating that diets rich in ruminant meat are among the most carbon- and water-intensive.

Substantial heterogeneity was observed across studies. The greater variability in the pooled carbon footprint observed in studies using food purchase surveys may stem from the limitations of these surveys in estimating actual food consumption. Household Consumption and Expenditure Surveys estimate apparent consumption rather than individual intake, lack information on intra-household distribution, often omit food consumed outside the home and rarely account for cooking processes^(52,82–84)^. These factors may result in systematic over- or underestimation of environmental impacts.

Sociodemographic differences further shaped findings. Higher diet-related GHGE among individuals with higher educational level and urban residence may reflect dietary patterns associated with higher income and food affordability. Urban populations in Brazil and Mexico, for example, tend to consume more beef and fewer legumes^(54,85)^. Meat remains among the most expensive components of household food baskets^(49,58)^, and omnivorous diets are generally more costly than plant-based alternatives^(48,54,86)^. Similar socioeconomic gradients in dietary environmental impact have been reported in high-income countries, suggesting that rising income may intensify environmental pressures in middle-income settings undergoing nutrition transition^(16,87)^.

Compared with studies from high-income countries, the range of carbon footprint estimates observed in Latin America was wider. While mean values reported in Europe, the United States and Australia generally range between approximately 3 and 6 kg CO₂eq per person per day^(14–16)^, our unstandardised estimates ranged from 2·2 kg CO₂eq in Peru to 8·3 kg CO₂eq in Argentina. Differences may partly reflect methodological variation, particularly the use of food purchase *v*. individual consumption surveys, which have been shown to introduce systematic differences and affect environmental impact estimates^(88)^. However, structural differences in dietary patterns, production systems and socioeconomic contexts are also likely contributors, as environmental footprints reflect diverse food systems and consumption drivers across regions^(89)^.

Overall, livestock consumption, particularly beef, emerges as the dominant driver of dietary environmental impact in the region. Yet limited geographic coverage, narrow environmental scope and methodological heterogeneity constrain the ability to draw comprehensive regional conclusions.

### Research gaps and future directions

First, geographic coverage remains insufficient. Nationally representative data are unavailable for most Latin American countries, limiting regional synthesis.

Second, environmental assessment remains largely confined to carbon and water footprints^(90)^. Broader indicators such as land-system change, eutrophication potential, fossil energy demand and biodiversity loss were rarely incorporated. These dimensions are central to planetary boundary frameworks and are particularly relevant in a region containing globally significant ecosystems^(13,90)^. Expanding multidimensional environmental assessments would improve alignment with global sustainability targets.

Third, methodological standardisation is urgently needed. Environmental impact estimates may be derived from international or national databases, and the use of country-specific production data improves contextual accuracy. Conversion factors reflecting edible portions and cooking transformations were inconsistently applied. Cooking-related emissions were included in some studies but omitted in others, despite substantial variation in household energy sources across the region. Incorporating country-specific energy profiles would allow more comprehensive assessment of food preparation emissions^(91,92)^.

Fourth, food loss and waste were not explicitly considered in most of the reviewed studies. Globally, approximately 14 % of food is lost between harvest and retail, and a further 19 % is wasted at retail and household levels^(93,94)^. The Intergovernmental Panel on Climate Change estimates that food loss and waste account for 8–10 % of total anthropogenic GHGE^(95)^. Failure to incorporate these losses may lead to systematic underestimation or overestimation of diet-related environmental impacts, particularly in studies based on household purchase data, which reflect apparent rather than consumed intake. In Latin America, where post-harvest infrastructure, cold-chain capacity and retail systems vary substantially across countries, incorporating context-specific food loss and waste coefficients would improve the accuracy and comparability of environmental footprint estimates. Future research should explicitly define system boundaries and integrate food loss and waste adjustments to better capture the full environmental burden of diets.

Fifth, future analyses should integrate environmental impact with dietary quality. In contexts where undernutrition and food insecurity persist, reducing environmental impact must not compromise nutritional adequacy^(11,81,96)^. Lower meat intake in some settings may reflect economic constraint rather than sustainable choice^(96)^. Integrated assessments that jointly evaluate environmental sustainability and nutritional quality are essential for equitable policy design^(97)^.

Finally, the environmental implications of ultra-processed foods remain underexplored. Although often discussed in relation to health outcomes, these products are embedded in industrial supply chains reliant on intensive monocultures, energy-intensive processing, packaging and long distribution networks^(4,98)^. As ultra-processed foods become increasingly dominant in regional diets, their combined health and environmental impacts warrant systematic evaluation^(99,100)^.

### Limitations and strengths

To the best of our knowledge, this is the first systematic review with meta-analysis to provide a comprehensive overview of research on the environmental impact of diets in Latin America. This work establishes an important precedent by identifying research gaps, highlighting methodological approaches for estimating dietary environmental footprints, and providing evidence to support future public policies aimed at promoting sustainable diets in the region.

However, some limitations should be considered. Our search strategy did not include grey literature, which may have led to the omission of relevant studies. Nevertheless, we conducted a thorough review of reference lists from all included studies, allowing us to identify and incorporate additional research. An additional limitation is the substantial heterogeneity observed in the pooled estimates of carbon footprint within the meta-analysis, despite the application of several predefined criteria for study inclusion. This level of heterogeneity was anticipated, considering the diverse demographic and cultural contexts of Latin American countries, as well as the methodological variability between studies. Nevertheless, no statistically significant differences were identified based on the type of dietary assessment employed. Moreover, the heterogeneity is unlikely to be attributable to the inclusion of studies with differing LCA boundaries, given that the initial stage of the LCA – spanning from food production to the farm gate (included in the EII estimates of all the studies) – accounts for approximately 80 % of food-related GHGE^(5)^. Therefore, it is plausible that the heterogeneity primarily reflects the intrinsic variability of dietary patterns across the region.

### Conclusion

Across eight Latin American countries with available data, the pooled energy-standardised dietary carbon footprint was 4·1 kg CO₂eq per person per day, with Argentina and Peru showing the highest and lowest estimates, respectively. Brazil exhibited the highest reported water footprint. Consumption of livestock products particularly beef, is the primary driver of dietary environmental impact, with notable variation across countries and sociodemographic groups.

Major gaps persist in geographic coverage, environmental scope and methodological harmonisation. Strengthening nationally representative dietary data systems, expanding multidimensional environmental assessments and integrating sustainability with nutritional adequacy will be critical to inform policies that promote equitable, healthy and environmentally sustainable food system in Latin America.

## Supporting information

10.1017/S1368980026102584.sm001 Araya-Bastias et al. supplementary material 1 Araya-Bastias et al. supplementary material

10.1017/S1368980026102584.sm002 Araya-Bastias et al. supplementary material 2 Araya-Bastias et al. supplementary material

10.1017/S1368980026102584.sm003 Araya-Bastias et al. supplementary material 3 Araya-Bastias et al. supplementary material

10.1017/S1368980026102584.sm004 Araya-Bastias et al. supplementary material 4 Araya-Bastias et al. supplementary material

## Supplementary material

For supplementary material accompanying this paper visit https://doi.org/10.1017/S1368980026102584
