# Supplementary material for: The environmental impact of diet in Latin American populations: a systematic review with meta-analysis
Source: Public Health Nutr. 2026 May 4;29(1):e125. doi: 10.1017/S1368980026102584 (PMC13434614; doi:10.1017/S1368980026102584)
Supplement: Supplementary file 1 [file S1368980026102584sup001.pdf]

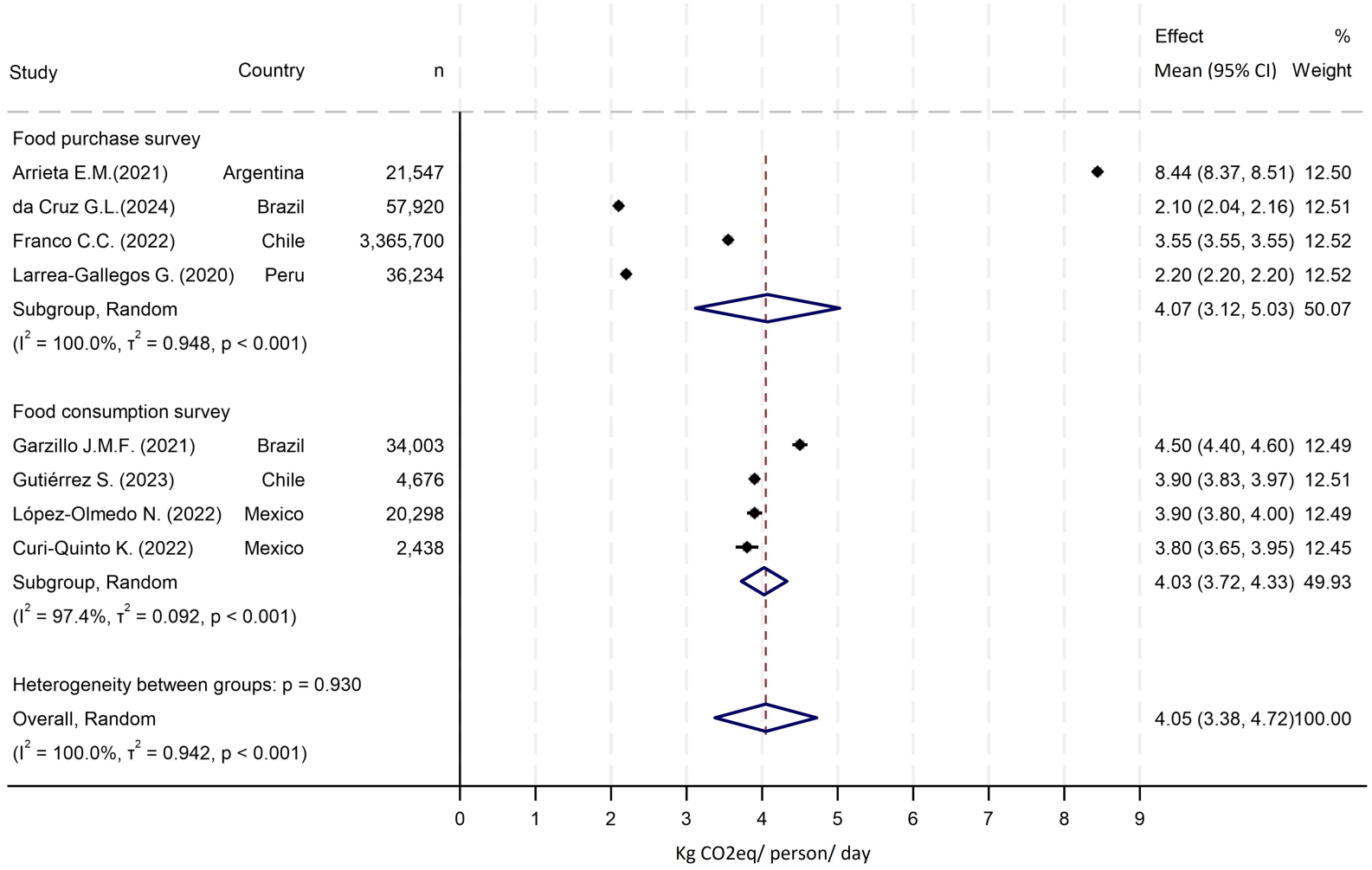
Study
Country
n
Effect
Mean (95% CI)
%
Weight
Food purchase survey
Arrieta E.M.(2021) Argentina 21,547 8.44 (8.37, 8.51) 12.50
da Cruz G.L.(2024) Brazil 57,920 2.10 (2.04, 2.16) 12.51
Franco C.C. (2022) Chile 3,365,700 3.55 (3.55, 3.55) 12.52
Larrea-Gallegos G. (2020) Peru 36,234 2.20 (2.20, 2.20) 12.52
Subgroup, Random 4.07 (3.12, 5.03) 50.07
(I^2 = 100.0%, τ^2 = 0.948, p < 0.001)
Food consumption survey
Garzillo J.M.F. (2021) Brazil 34,003 4.50 (4.40, 4.60) 12.49
Gutiérrez S. (2023) Chile 4,676 3.90 (3.83, 3.97) 12.51
López-Olmedo N. (2022) Mexico 20,298 3.90 (3.80, 4.00) 12.49
Curi-Quinto K. (2022) Mexico 2,438 3.80 (3.65, 3.95) 12.45
Subgroup, Random 4.03 (3.72, 4.33) 49.93
(I^2 = 97.4%, τ^2 = 0.092, p < 0.001)
Heterogeneity between groups: p = 0.930
Overall, Random 4.05 (3.38, 4.72)100.00
(I^2 = 100.0%, τ^2 = 0.942, p < 0.001)
0 1 2 3 4 5 6 7 8 9
Kg CO2eq/ person/ day
