# Supplementary material for: The environmental impact of diet in Latin American populations: a systematic review with meta-analysis
Source: Public Health Nutr. 2026 May 4;29(1):e125. doi: 10.1017/S1368980026102584 (PMC13434614; doi:10.1017/S1368980026102584)
Supplement: Supplementary file 2 [file S1368980026102584sup002.pdf]

Search terms by database

Medline:

1. central america/ or latin america/ or south america/

2. Latin America*.tw.

3. (Argentin* or Belize* or Bolivia* or Brazil* or Chile* or Colombia* or Costa Rica* or Ecuador* or El Salvador or Guatemala* or Guyan* or Honduras or Paraguay* or Mexic* or Nicaragua* or Panama* or Peru* or Suriname* or Uruguay* or Venezuela*).ti,ab,in,ab.

4. or/1-3

5. environment/ or carbon footprint/ or fresh water/ or soil/ or eutrophication/

6. carbon dioxide/ or greenhouse gases/

7. Greenhouse Effect/

8. ((environment* or greenhouse gas* or carbon) adj2 (impact or footprint or emission*)).tw.

9. Fresh Water/

10. water/ or drinking water/

11. ((Fresh water or freshwater or water) adj2 (us* or consum* or footprint or stress* or scarc*)).tw.

12. agriculture/ or "food and beverages"/

13. ((Land or cropland* or ecological or agricultural) adj2 (us* or footprint or occupation or demand)).tw.

14. or/5-13

15. 4 and 14

16. Diet/

17. Diet Surveys/

18. Eating/

19. Food/

20. united nations food.mp. and agricultural organization.ti,ab,in,ab. [mp=title, book title, abstract, original title, name of substance word, subject heading word, floating sub-heading word, keyword heading word, organism supplementary concept word, protocol supplementary concept word, rare disease supplementary concept word, unique identifier, synonyms, population supplementary concept word, anatomy supplementary concept word]

21. ((diet* or eating or food* or apparent food or FAO food balance sheet) adj2 (pattern* or intake or consum* or purchase)).tw.

22. or/16-21

23. 15 and 22

Embase:

1. exp "South and Central America"/

2. Latin America*.tw.

3. (Argentin* or Belize* or Bolivia* or Brazil* or Chile* or Colombia* or Costa Rica* or Ecuador* or El Salvador or Guatemala* or Guyan* or Honduras or Paraguay* or Mexic* or Nicaragua* or Panama* or Peru* or Suriname* or Uruguay* or Venezuela*).ti,ab,in,ab.

4. or/1-3

5. environmental impact/ or environmental erosion/ or eutrophication/ or greenhouse effect/ or "human impact (environment)"/ or overexploitation/

6. methane/ or greenhouse effect/ or carbon dioxide/ or greenhouse gas/ or greenhouse/

7. exp carbon footprint/

8. ((environment* or greenhouse gas* or carbon) adj2 (impact or footprint or emission*)).tw.

9. exp fresh water/

10. water/ or drinking water/

11. ((Fresh water or freshwater or water) adj2 (us* or consum* or footprint or stress* or scarc*)).tw.

12. exp land footprint/ or exp agricultural land/

13. ((Land or cropland* or ecological or agricultural) adj2 (us* or footprint or occupation or demand)).tw.

14. or/5-13

15. 4 and 14

16. diet/

17. diet* survey*.ti,ab,in,ab.

18. eating/

19. food/

20. exp "united nations food and agricultural organization"/

21. ((diet* or eating or food* or apparent food or FAO food balance sheet) adj2 (pattern* or intake or consum* or purchase)).tw.

22. or/16-21

23. 15 and 22

Web of Sciences:

Search: TS=(Argentin* or Belize* or Bolivia* or Brazil* or Chile* or Colombia* or "Costa Rica*" or Ecuador* or "El Salvador" or Guatemala* or Guyan* or Honduras or Paraguay* or Mexic* or Nicaragua* or Panama* or Peru* or Suriname* or Uruguay* or Venezuela*)

Search: TS=((environment* or "greenhouse gas*" or carbon) near/2 (impact or footprint or emission))

Search: TS=(("Fresh water" or freshwater or water) near/2 (use or usage or consum* or stress* or scarc* or footprint))

Search: TS=((Land or cropland* or ecological or agricultural) near/2 (use or usage or footprint or occupation or demand))

Search: #2 OR #3 OR #4

Search: #1 AND #5

Search: TS=((diet* or eating or food* or "apparent food" or "FAO food balance sheet") near/2 (pattern* or intake or consum* or purchase))

Search: #6 AND #7

Scopus

(TITLE-ABS-KEY (argentin* OR belize* OR bolivia* OR brazil* OR chile* OR colombia* OR "Costa Rica*" OR ecuador* OR "El Salvador" OR guatemala* OR guyan* OR honduras OR paraguay* OR mexic* OR nicaragua* OR panama* OR peru* OR suriname* OR uruguay* OR venezuela*) AND TITLE-ABS-KEY ((environment* OR "greenhouse gas*" OR carbon W/2 impact OR footprint OR emission*) OR ("Fresh water" OR freshwater OR water W/2 use OR usage OR consum* OR stress* OR scarc* OR footprint) OR (land OR cropland* OR ecological OR agricultural W/2 use OR usage OR footprint OR occupation OR demand)) AND TITLE-ABS-KEY (diet* OR eating OR food* OR "apparent food" OR "FAO food balance sheet" W/2 pattern* OR intake OR consum* OR purchase))
